# Effects of cataract surgery in Japanese patients with neovascular age-related macular degeneration

**DOI:** 10.1007/s00417-020-05015-w

**Published:** 2020-11-18

**Authors:** Fumi Nishiguchi, Hiroto Ishikawa, Junichi Amaki, Yuki Komuku, Akiko Kimura, Fumi Gomi

**Affiliations:** Department of Ophthalmology, Hyogo College of Medicine, 1-1 Mukogawa-cho, Nishinomiya, Hyogo 663-8501 Japan

**Keywords:** Patient satisfaction, Age-related macular degeneration, Cataract surgery, Intraocular lens, Postoperative survey, Metamorphopsia

## Abstract

**Purpose:**

To investigate the effects of cataract surgery in Japanese patients with neovascular age-related macular degeneration (nAMD).

**Methods:**

The primary endpoint in this prospective, observational study was patient satisfaction at 6 months after cataract surgery in patients with nAMD. Secondary endpoints comprised changes in best-corrected visual acuity (BCVA), M-chart score, central retinal thickness (CRT), AMD status, and number of AMD treatments. All examinations were performed before surgery, and at 1, 3, and 6 months postoperatively.

**Results:**

Fifty patients (52 eyes) were included in this study (32 men; mean age, 76.1 ± 7.1 years). Thirty-nine patients (75.0%) reported satisfaction with cataract surgery. BCVA significantly improved at all postoperative timepoints (all *p* < 0.0001), whereas differences in M-chart scores were not statistically significant. The number of eyes with BCVA ≤ 0.3 logarithm of the minimum angle of resolution (logMAR) increased from 21 to 38; however, CRT did not change. The number of AMD treatments did not change during follow-up. All questionnaire scores showed postoperative improvement. Univariate and multivariate analyses revealed that final BCVA ≤ 0.3 logMAR was significantly associated with patient satisfaction.

**Conclusion:**

Cataract surgery significantly improved vision in Japanese patients with nAMD, without affecting AMD status. Patients were satisfied with cataract surgery, especially with respect to improvement of distance vision.

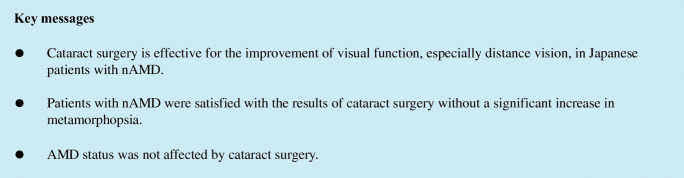

## Introduction

In 2019, the United Nations reported that there were 703 million persons aged ≥ 65 years worldwide; of these, 261 million were in southeastern Asia [1]. With regard to visual health, aging is an important factor in the onset of both cataracts and age-related macular degeneration (AMD).

Cataracts are the leading cause of blindness worldwide, while AMD is the third leading cause [2]. Cataracts gradually interfere with a patient’s vision [2, 3], while neovascular AMD (nAMD) causes sudden vision loss. Symptoms of nAMD include central vision reduction, metamorphopsia, and general haziness [4–7].

With regard to treatments, cataract surgery is generally regarded as safe and effective [8, 9]. For treatment of nAMD, intravitreal anti-vascular endothelial growth factor (VEGF) injection and photodynamic therapy are common approaches with robust supporting evidence [10–12]. Aging contributes to both cataracts and nAMD; thus, some patients require both therapies concurrently.

Cataract surgery is reportedly effective for the improvement of visual function in patients with AMD [13–17]. Because sunlight (ultraviolet (UV) light) is a risk factor [18], it may be beneficial to use UV light-blocking intraocular lenses (IOLs) in older patients who undergo cataract surgery. To the best of our knowledge, there have been no studies regarding the efficacy of cataract surgery in Japanese patients with nAMD.

In the present study, we aimed to examine the efficacy of cataract surgery in Japanese patients with nAMD, by assessing changes in visual function and retinal anatomy, as well as by means of a customized questionnaire regarding patient satisfaction.

## Materials and methods

### Study design and eligibility

This was a prospective study of 54 patients with nAMD who had undergone cataract surgery between December 2016 and June 2019. Written informed consent was obtained from all participants. The current study was performed in accordance with the Declaration of Helsinki and with approval from the ethics committee of Hyogo College of Medicine (approval no. 2494).

### Patients

Inclusion criteria were age > 50 years, simultaneous diagnosis of cataract and AMD, and no history of intraocular surgery (excluding intravitreal anti-VEGF injections) in the eye undergoing surgery. Exclusion criteria were diagnosis of diabetic retinopathy, retinal vein or artery occlusion, high myopia (i.e., axial length > 28 mm), and/or any type of glaucoma. Finally, 56 eyes of 54 patients were recruited for this study.

### Surgical procedure

All patients had undergone cataract surgery using the same type of UV light-blocking IOLs (PCB00V; AMO Japan, Tokyo, Japan); surgeries were performed by two ophthalmologists (FG and HI). All patients underwent normal phacoemulsification and aspiration with IOL insertion (PEA + IOL) using a cataract surgery phacoemulsification device (Centurion Vision System, Alcon Japan, Tokyo, Japan).

### Study protocol

The following demographic and clinical data were evaluated in this study: age, sex, BCVA, metamorphopsia, central retinal thickness (CRT), AMD status and subtype, number of treatments for AMD before and after cataract surgery, and satisfaction questionnaire results. Decimal visual acuity was examined using the Landolt C chart with the following consistent conditions: distance, 5 m; chart illuminance, 500–1000 lux. The decimal visual acuity was then converted to logarithm of the minimum angle of resolution (logMAR) values for statistical analysis. A change in BCVA of one line was regarded as a change of 0.1 logMAR. The degree of metamorphopsia was quantified by using the M-chart tool (Inami, Tokyo, Japan), and the sum of vertical and horizontal M-chart scores was used for analysis.

AMD subtype was determined by one ophthalmologist (FG) using fundus photos, optical coherence tomography, and fundus fluorescein/indocyanine angiography during the initial examination for treatment of AMD. Regarding AMD status, eyes with “active” AMD were those that had received any AMD treatments within 6 months before cataract surgery. Decisions concerning AMD treatment were made by the two ophthalmologists (FG and HI) on the basis of routine clinical practice and each patient’s disease status. Treatment mostly comprised a pro re nata regimen, according to the presence of subretinal fluid, hemorrhage, and any exudation on fundus photos and optical coherence tomography scans. The number of AMD treatments was defined as the sum of the number of intravitreal anti-VEGF injections and photodynamic therapy applications, compared between 6 months before and 6 months after cataract surgery. Patient satisfaction was assessed by the independent investigators using a customized questionnaire (Table 1), both preoperatively and at 6 months postoperatively. The preoperative questionnaire had seven questions, while the postoperative questionnaire had eight questions. The score for each question was as follows: 1, very good; 2, good; 3, fair; 4, poor; and 5, very poor.

**Table 1:** Customized questionnaire used in this study

		1	2	3	4	5
Q1.	How much difficulty do you have reading street signs or the names of stores?	Very good	Good	Fair	Poor	Very poor
Q2.	How much difficulty do you have watching TV?	Very good	Good	Fair	Poor	Very poor
Q3.	How much difficulty do you have using a personal computer or cooking?	Very good	Good	Fair	Poor	Very poor
Q4.	How much difficulty do you have reading ordinary print in newspapers/books/magazines?	Very good	Good	Fair	Poor	Very poor
Q5.	Do you experience double vision?	Very good	Good	Fair	Poor	Very poor
Q6.	Do you experience halos and glare?	Very good	Good	Fair	Poor	Very poor
Q7.	How much difficulty do you have seeing clearly at night?		Good	Fair	Poor	
Q8.	Are you satisfied with the cataract surgery that you received?	Very satisfied	Satisfied	Neutral	Dissatisfied	Very dissatisfied

Patients were followed up for > 6 months postoperatively. Ophthalmological examinations were performed preoperatively, as well as at 1, 3, and 6 months postoperatively. Changes in BCVA, M-chart score, CRT, number of AMD treatments, and questionnaire results were then compared between baseline and postoperative follow-up examinations.

### Study endpoints

The primary endpoint was patient satisfaction after cataract surgery in Japanese patients with nAMD. Secondary endpoints comprised changes in BCVA, M-chart score, CRT, AMD status, number of AMD treatments, and postoperative complications.

### Statistical analyses

For continuous variables, the mean, standard deviation, and range were calculated. For categorical variables, the numbers and percentages of values in each category were calculated. Student’s *t* test or the Wilcoxon signed-rank test was used to assess group differences in continuous variables; Fisher’s exact test or the Pearson *χ*^2^ test was used to assess group differences in categorical variables. Analyses were performed using JMP® Pro (version 14.0.0, SAS Institute Inc., Cary, NC, USA). For all analyses, *p* values were reported; two-sided 95% confidence intervals were also reported for point estimates. *p* < 0.05 was considered to indicate statistical significance.

## Results

### Baseline characteristics

**Fig. 1:**
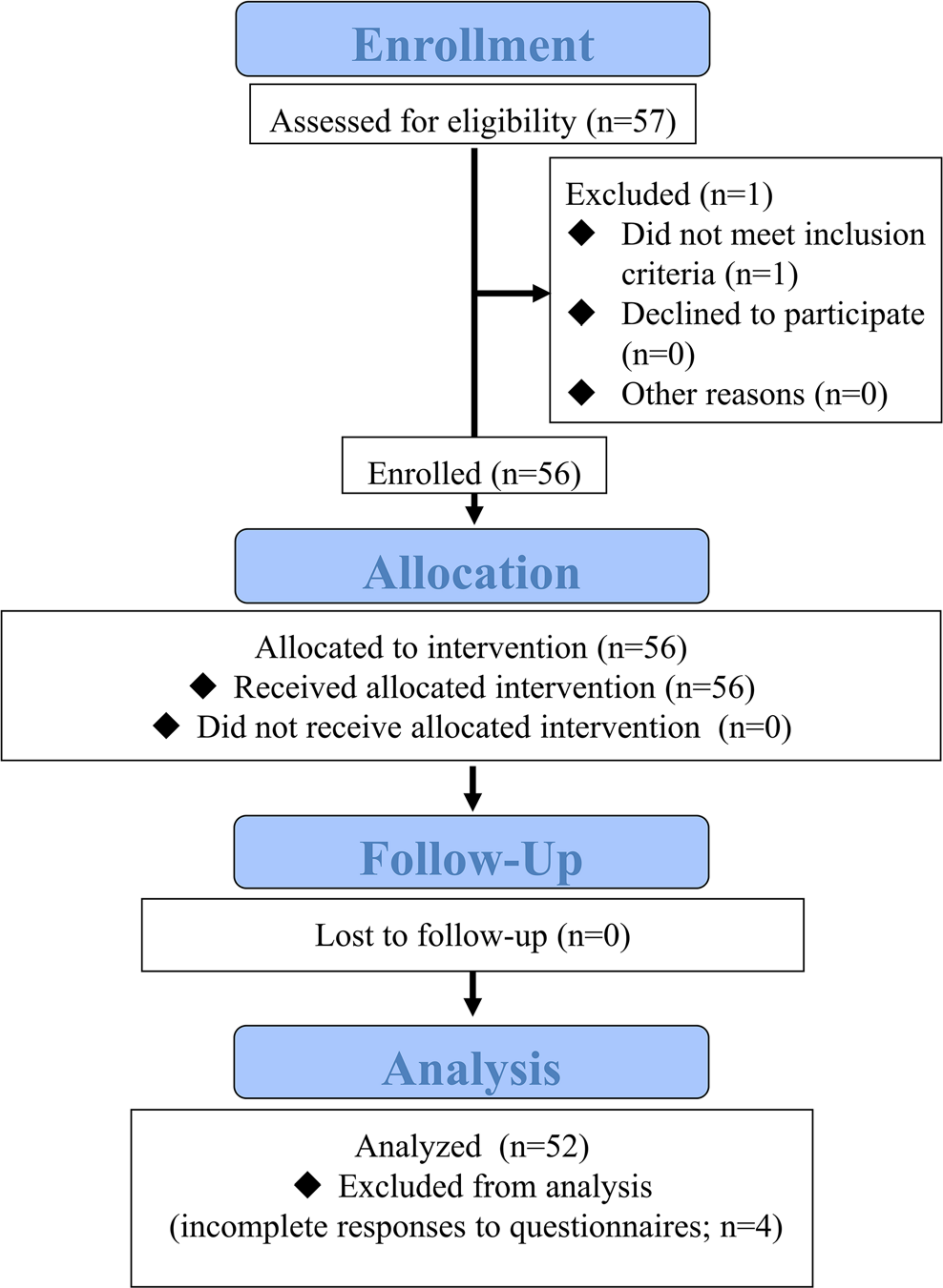
CONSORT flow diagram. A total of 54 patients (56 eyes) were enrolled. Four patients (four eyes) provided incomplete responses to questionnaires. Therefore, 50 patients (52 eyes) were included in the analysis

The CONSORT flow diagram is shown in Fig. 1. Overall, 54 patients (56 eyes) were enrolled in this study and underwent cataract surgery. Four patients (four eyes) provided incomplete responses to questionnaires. Therefore, 50 patients (52 eyes) were included in the analysis. Patients were predominantly men (*n* = 32, 64.0%) with a mean age of 76.1 ± 7.1 years. All patients had both AMD and cataracts. Bilateral AMD was present in two patients. AMD status was active in 18 eyes (34.6%). Regarding AMD subtype, typical AMD, polypoidal choroidal vasculopathy (PCV), and retinal angiomatous proliferation were found in 27, 24, and 1 patients, respectively. The mean (± standard deviation) BCVA at baseline was 0.43 ± 0.40 logMAR (range, − 0.079 to 1.699 logMAR); 43 eyes (82.7%) had worse BCVA, compared with fellow eyes. Eyes with PCV had significantly better BCVA (0.26 ± 0.27 logMAR) than those with typical AMD (0.57 ± 0.45 logMAR) (*p* = 0.0034). The mean M-chart score at baseline was 0.92 ± 1.08 (range, 0–4.0). The mean axial length was 23.66 ± 1.19 mm (range, 20.73–26.78 mm).

### Primary endpoint: patient satisfaction

**Fig. 2:**
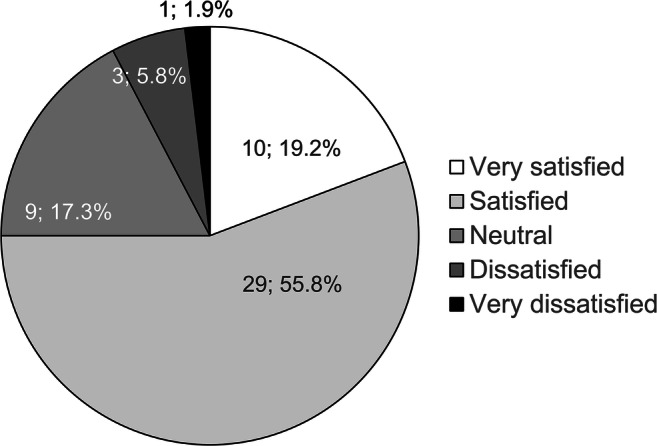
Patient satisfaction findings. Among patients in this study, 75.0% were satisfied with cataract surgery

**Fig. 3:**
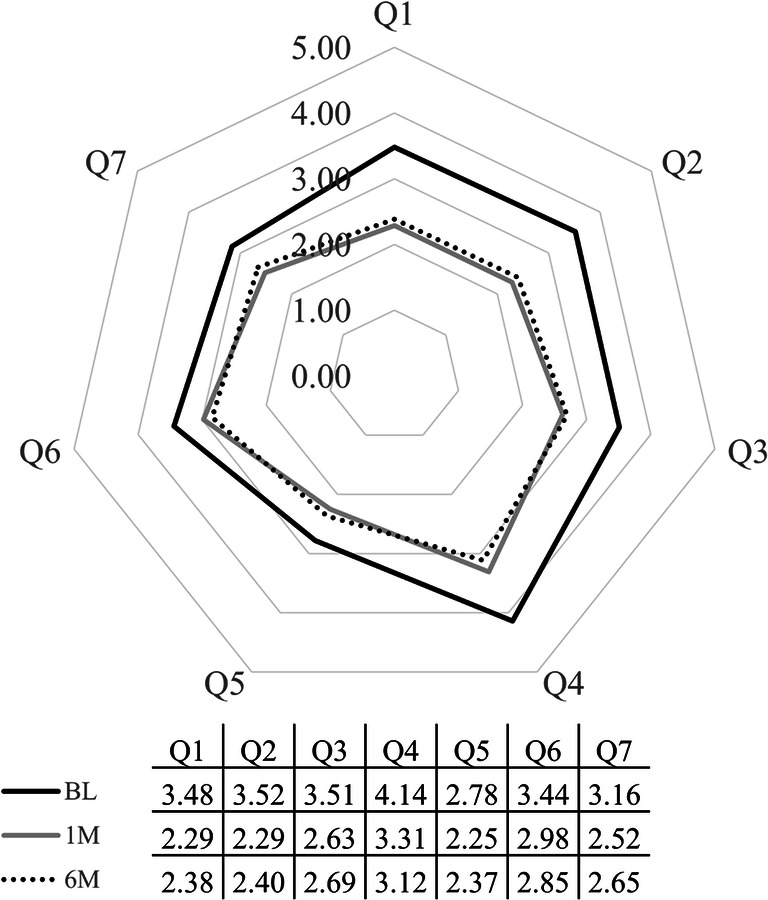
Questionnaire results. All scores showed postoperative reduction; Q1, Q2, and Q4 each exhibited reductions of > 1 point

Patient satisfaction results are shown in Fig. 2. Thirty-nine patients (75.0%) reported satisfaction (“very satisfied” + “satisfied”) with cataract surgery. The questionnaire results are shown in Fig. 3. All scores showed reduction postoperatively; Q1, Q2, and Q4 each exhibited reductions of > 1 point. However, the questionnaire scores showed that most patients encountered similar difficulty reading ordinary print in newspapers/books/magazines, when compared between before and after cataract surgery.

### Secondary endpoints: postoperative changes in several parameters

Changes in several parameters regarding secondary endpoints are shown in Tables 2 and 3. BCVA significantly improved at all postoperative timepoints (all *p* < 0.0001), whereas mean M-chart scores did not significantly change at any postoperative timepoints, although the scores increased slightly. At 6 months postoperatively, BCVA improvement of ≥ 2 lines was observed in 24 eyes (46.2%) and the number of eyes with BCVA ≤ 0.3 logMAR increased from 21 (40.4%) to 38 (73.1%). Regarding AMD subtype, the proportion of eyes with BCVA ≤ 0.3 logMAR was higher among eyes with PCV than among eyes with typical AMD throughout the follow-up period. CRT did not change at any postoperative timepoints (Table 2).

**Table 2:** Changes in several parameters regarding secondary endpoints

	Preoperative examination	1-month postoperative examination	*p* value*	3-month postoperative examination	*p* value#	6-month postoperative examination	*p* value†
Mean BCVA (logMAR)	0.43 ± 0.40	0.25 ± 0.33	***< 0.0001***	0.24 ± 0.38	***< 0.0001***	0.24 ± 0.36	***< 0.0001***
Number of eyes with logMAR BCVA ≤ 0.3							
Total	21 (40.4%)	35 (67.3%)	***0.0004***	38 (73.1%)	***0.0030***	38 (73.1%)	***0.0030***
Typical AMD	7 (25.9%)	15 (55.6%)	0.0621	17 (63.0%)	***0.0184***	17 (63.0%)	***0.0184***
PCV	14 (58.3%)	20 (83.3%)	***0.0095***	21 (87.5%)	0.3478	21 (87.5%)	0.3478
RAP	0	0		0		0	
Number of eyes with BCVA improvement > logMAR 0.2							
Total		25 (48.0%)		27 (51.9%)		24 (46.2%)	
Typical AMD		15 (55.6%)		17 (63.0%)		15 (55.6%)	
PCV		9 (37.5%)		10 (41.7%)		9 (37.5%)	
RAP		1 (100%)		0		0	
M-chart score	0.92 ± 1.08	0.78 ± 0.98	0.2404	1.06 ± 1.28	0.5316	1.05 ± 1.25	0.2893
CRT	281.1 ± 124.5	273.4 ± 118.6	0.9106	283.34 ± 138.1	0.7670	300.4 ± 149.2	0.2118

*BCVA*, best-corrected visual acuity; *logMAR*, logarithm of the minimum angle of resolution; *AMD*, age-related macular degeneration; *PCV*, polypoidal choroidal vasculopathy; *RAP*, retinal angiomatous proliferation; *CRT*, central retinal thickness

Bold italic font indicates statistical significance (*p* < 0.05)

*Pairwise comparison between preoperative and 1-month postoperative examinations

#Pairwise comparison between preoperative and 3-month postoperative examinations

†Pairwise comparison between preoperative and 6-month postoperative examinations

**Table 3:** Changes in AMD status

	Preoperative 6 months	Postoperative 6 months	*p* value
AMD status			
Number of eyes with active AMD	35/52 67.3%	27/52 51.9%	***0.0043***
Number of AMD treatments in total participants	1.2 ± 1.1	0.9 ± 1.0	0.1002
PDT	0.1 ± 0.3	0.1 ± 0.2	0.4195
Anti-VEGF	1.1 ± 1.1	0.8 ± 1.0	0.1106
Number of AMD treatments in participants with active AMD	1.7 ± 1.0	1.4 ± 1.0	***0.0046***
PDT	0.1 ± 0.2	0	0.1817
Anti-VEGF	1.6 ± 1.0	1.4 ± 1.0	***0.0139***

*AMD*, age-related macular degeneration; *PDT*, photodynamic therapy; *VEGF*, vascular endothelial growth factor

Bold italic font indicates statistical significance (*p* < 0.05)

The AMD status and details of AMD treatment are shown in Table 3. The number of eyes with active AMD during 6 months before and 6 months after cataract surgery were 35 (67.3%) and 27 (51.9%); this number was significantly lower after cataract surgery. However, four of 17 eyes without preoperative AMD treatments required postoperative AMD treatments. The number of AMD treatments did not change, compared between 6 months preoperatively and 6 months postoperatively. Intraoperative and postoperative complications were not observed in any patients.

### Univariate and multivariate analysis of associations with the primary endpoint

Univariate analysis revealed that the results of Q1 and Q2 at 6 months postoperatively, as well as final BCVA ≤ 0.3 logMAR, were significantly associated with patient *satisfaction (Q1: p* < 0.0001, Q2: *p* < 0.0001, final BCVA: *p* = 0.0006). Multivariate analysis of significantly associated parameters in univariate analysis revealed that only final BCVA ≤ 0.3 logMAR was significantly associated with patient satisfaction (*p* = 0.0026).

### Subgroup analysis: nine better-seeing eyes versus 43 worse-seeing eyes at baseline

Final BCVA ≤ 0.3 logMAR was achieved in nine (100%) better-seeing eyes and in 29 (67.4%) worse-seeing eyes (*p* = 0.0452). Changes in BCVA between baseline and 6 months were not significantly different, when compared between better-seeing eyes (− 0.12 ± 0.20 logMAR) and worse-seeing eyes (− 0.21 ± 0.24 logMAR).

Patient satisfaction was reported by eight (88.9%) patients with better-seeing eyes and 31 (72.1%) patients with worse-seeing eyes; this difference was not statistically significant.

## Discussion

In the present study, we demonstrated the efficacy of cataract surgery in Japanese patients with nAMD. More than three quarters of patients were satisfied with their postoperative quality of vision. BCVA was improved postoperatively, while metamorphopsia did not change. Anatomical changes in the retina, AMD status, number of AMD treatments, and CRT did not change during the follow-up period.

The patients in this study had fairly severe cataracts; therefore, an increase in BCVA was expected after cataract surgery. We presumed that patients would experience substantially greater metamorphopsia after surgery, because cataract removal could provide clearer vision [6]. Metamorphopsia is a hallmark symptom of AMD; the most common subjective symptoms comprise distortion of lines and features [19]. However, in the present study, most patients did not report substantial postoperative worsening of metamorphopsia, although it occurred in some patients. After cataract surgery, patients found it easier to recognize changes in their visual symptoms due to clearer vision, and at the same time physicians found it easier to monitor fundus changes through the clear media. Therefore, cataract surgery can be beneficial in the management of AMD.

AMD status, CRT, and the number of AMD treatments did not change during the follow-up period. These results suggested that cataract surgery did not affect AMD status. UV light-blocking IOLs were used in the present study. In a previous basic research study, blue-violet light exposure led to enhanced VEGF production in an in vitro model of AMD, suggesting that blue-violet light could enhance the retinal level of VEGF and damage retinal pigment epithelium cells [20]. UV light-blocking IOLs provide older adults with improved rhodopsin and melanopsin sensitivity, which might contribute to prevention of AMD [21, 22].

Regarding AMD subtype, the proportion of eyes that had postoperative BCVA ≤ 0.3 logMAR was significantly greater among eyes with PCV than among eyes with typical AMD. This was presumably because vision was better at baseline in eyes with PCV than in eyes with typical AMD. It has been well-known that patients with PCV are relatively younger and have better visual prognosis than typical AMD, and cataract surgery might prove more beneficial in PCV patients in terms of the better vision over the course of their lives [23, 24].

The results of all questionnaire questions were improved; in particular, questions regarding far vision (Q1) and middle-range vision (Q2) revealed considerable improvement. Q1, Q2, and final BCVA ≤ 0.3 logMAR were significantly associated with patient satisfaction in univariate analysis. These three factors are related to middle–far vision. Patients with nAMD experience difficulty reading books or newspapers (using near vision) due to central scotoma; notably, cataract surgery can aid in middle–far vision, but not near vision.

Multivariate analysis revealed that only final BCVA ≤ 0.3 logMAR was associated with patient satisfaction. Thus, final BCVA ≤ 0.3 logMAR is important for patient satisfaction after cataract surgery. We previously reported that in patients with AMD who had final BCVA ≤ 0.3 logMAR or improvements of > 15 letters after anti-VEGF therapy exhibited greater improvements in National Eye Institute Visual Function Questionnaire-25 composite score, compared with patients who did not experience BCVA improvements; this finding suggested that better final BCVA was important for vision quality, regardless of the method to achieve improved BCVA [12].

In subgroup analysis of better-seeing and worse-seeing eyes at baseline, the number of eyes with final BCVA ≤ 0.3 logMAR was significantly different between the two groups. These results indicated that when the operated eyes exhibited better vision than fellow eyes, patients tended to experience greater satisfaction with BCVA improvement.

In a population-based case-control study in Taiwan regarding the association between AMD and dementia, the risk of dementia onset was greater in patients with AMD, relative to patients without AMD. Moreover, the risk was greater in patients who did not receive cataract surgery than in patients who did receive surgery [25]. When considered with these previous results, the current findings suggest that the risk of dementia in patients with AMD could be reduced by undergoing cataract surgery. Maintenance of visual function by adequate management of eyes with AMD could provide better quality of life for older adults.

There were several limitations in the present study. First, this study received financial support from AMO Japan and used IOLs from this company alone; however, we presume that this did not affect the results. Second, this study included a small number of patients with various stages of AMD; thus, it was difficult to determine whether the visual improvement was due to cataract surgery, AMD treatment, or both. Accordingly, the findings of the study should be confirmed in a larger cohort of patients with different types of IOLs.

In conclusion, the results of this study suggest that cataract surgery outcomes were satisfactory for Japanese patients with nAMD. We recommend performing cataract surgery in patients with AMD when their cataracts have become clinically significant.

## Data Availability

The data can be obtained with a request to the corresponding author. There is no confidential data or any restriction on accessing to the original data.
